# Next-Generation Electronics and Sensing Technology

**DOI:** 10.3390/s21237958

**Published:** 2021-11-29

**Authors:** Trong-Yen Lee, Yen-Lin Chen, Yu-Cheng Fan

**Affiliations:** 1 Department of Electronic Engineering, National Taipei University of Technology, Taipei 10608, Taiwan; tylee@mail.ntut.edu.tw; 2 Department of Computer Science and Information Engineering, National Taipei University of Technology, Taipei 10608, Taiwan; ylchen@mail.ntut.edu.tw

This Special Issue is dedicated to several aspects of next-generation electronics and sensing technology and contains eight papers that focus on advanced sensing devices, sensing systems, and sensing circuits that focus on the state-of-the-art methods for sensing technologies. These emerging sensing technologies will describe the future vision and trends for scholars and researchers. New sensing devices, circuits and systems technologies represent an important direction for electronics development. Due to these reasons, the main objectives of this Special Issue of the IEEE International Symposium on Next-Generation Electronics addresses novel sensing technologies. We rigorously selected and published the eight best papers in *Sensors*, a leading and important international journal in this field. Three important sensor-related themes are presented and discussed, including next-generation sensing devices, advanced sensing systems, and emerging sensing circuits. These proposed schemes are innovative and revolutionary and will be described in detail below.

The studies [1,2,3,4] propose next-generation sensing devices. In [1], Chen et al. designed a steep switching of In_0.18_Al_0.82_N/AlN/GaN MIS-HEMT (Metal Insulator Semiconductor High Electron Mobility Transistors) on Si for sensor applications [1]. InAlN/Al/GaN HEMTs on Si with a dynamic threshold voltage for a steep subthreshold slope (<60 mV/dec) are demonstrated in this study and attributed to displacement charge transition effects [1]. A material analysis with High-Resolution X-ray Diffraction (HR-XRD) and relaxation by reciprocal space mapping (RSM) were performed to confirm the indium barrier composition and epitaxy quality [1]. The proposed InAlN barrier HEMTs exhibit a high ON/OFF ratio with seven magnitudes, and a steep threshold swing (SS) was also obtained, with an SS = 99 mV/dec for the forward sweep and SS = 28 mV/dec for the reverse sweep [1]. GaN-based HEMTs on Si exhibited an outstanding performance in this study, with a high ON/OFF ratio and an SS < 60 mV/dec [1].

Liu et al. presented the fabrication and characterization of a planar-type top-illuminated InP-based avalanche photodetector on a conductive substrate with operating speeds exceeding 10 Gbps [2]. The design is based on a separate absorption, grading, charge, and multiplication (SAGCM) epitaxial structure [2]. An electric field profile of the SAGCM layers was derived from the epitaxial structure [2]. The punch-through voltage of the SAGCM APD was controlled to within 16–17 V, whereas the breakdown voltage (V_BR_) was controlled to within 28–29 V [2]. The design obtained a dark current of 2.99 nA, capacitance of 0.226 pF, and multiplication gain of 12 when the APD was biased at 0.9 V_BR_ at room temperature [2]. The frequency response was characterized by comparing the calculated 3 dB cut-off modulation frequency (f_3-dB_) and f_3-dB_ values measured under various multiplication gains and modulated incident powers [2]. The time response of the APD was evaluated by deriving eye diagrams at 0.9 V_BR_ using pseudorandom non-return-to-zero codes with a length of 2^31^–1 at 10–12.5 Gbps [2]. There was a notable absence of intersymbol interference, and the signals remained error-free at data rates of up to 12.5 Gbps [2]. The correlation between the rise time and modulated bandwidth demonstrate the suitability of the proposed SAGCM-APD chip for applications involving an optical-receiver at data rates of >10 Gbps [2]. 

The electrical and physical characteristics of a WO_3_/Ag/WO_3_ sandwich structure fabricated with magnetic-control sputtering metrology are analyzed in [3]. Three layers of transparent conductive films of WO_3_/Ag/WO_3_ (WAW) were deposited on a glass substrate by radio frequency (RF) magnetron sputtering in this work [3]. The thicknesses of WO_3_ (around 50~60 nm) and Ag (10~20 nm) films were the main changeable factors for achieving an optimal transparent conductivity when attempting to replace indium tin oxide (ITO) for cost considerations [3]. The prepared films were cardinally subjected to physical and electrical characteristic analyses by means of X-ray diffraction analysis (XRD), a field-emission scanning electron microscope (FE-SEM), and a Keithley 4200 semiconductor parameter analyzer [3]. The experimental results show that as the thickness of the Ag layer increases from 10 nm to 20 nm, the resistance becomes smaller [3]. Meanwhile, as the thickness of the WO_3_ layer increases from 50 nm to 60 nm, its electrical resistance becomes larger [3]. 

Chen and Wu addressed the sensing and reliability improvements of a transient electrostatic discharge by discrete engineering for high-voltage 60 V n-channel lateral-diffused MOSFETs with embedded silicon-controlled rectifiers [4]. High-voltage n-channel lateral-diffused metal–oxide–semiconductor field-effect transistor (nLDMOS) components, fabricated by a TSMC 0.25 μm 60-V bipolar-CMOS-DMOS (BCD) process with a drain-side embedded silicon-controlled rectifier (SCR) of the *n-p-n*-arranged and *p-n-p*-arranged types, were investigated in order to determine the devices’ electrostatic discharge (ESD)-sensing behaviors and capability by discrete anode engineering [4]. As for the drain-side *n-p-n*-arranged type with a discrete anode, the transmission-line pulse (TLP) testing results showed that the ESD ability (*I*_*t*2_ value) was slightly upgraded [4]. When the discrete physical parameter was 91 rows, the optimal *I*_*t*2_ reached 2.157 A (increasing by 17.7% when compared with the reference sample) [4]. On the other hand, the drain-side SCR *p-n-p*-arranged type with a discrete anode exhibited an excellent SCR behavior, and its *I*_*t*2_ values could be increased to >7 A (increasing by >281.9% when compared with the reference DUT) [4]. Moreover, under discrete anode engineering, the drain-side SCR *n-p-n*-arranged and *p-n-p*-arranged types had a clearly higher ESD ability, with the exception of the few discrete physical parameters [4]. Therefore, when using discrete-anode engineering, the ESD dissipation ability of a high-voltage (HV) nLDMOS with drain-side SCRs will have a greater effectiveness [4].

The studies [5,6] design advanced sensing systems. Chen et al. developed a learning-directed dynamic voltage and frequency scaling scheme with an adjustable performance for single-core and multi-core embedded and mobile systems [5]. This paper proposes a lightweight learning-directed dynamic voltage and frequency scaling (DVFS) method that involves using counter-propagation networks to sense and classify the task behavior and predict the best voltage/frequency setting for the system [5]. An intelligent adjustment mechanism for the performance is also provided to users under various performance requirements [5]. The comparative experimental results of the proposed algorithms and other competitive techniques are evaluated on the NVIDIA JETSON Tegra K1 multi-core platform and Intel PXA270 embedded platforms [5]. The results demonstrate that the learning-directed DVFS method can accurately predict the suitable central processing unit (CPU) frequency, given the runtime statistical information of a running program, and achieve an energy savings rate of up to 42% [5]. Through this method, users can easily achieve an effective energy consumption and performance by specifying the factors of performance loss [5]. 

Lee et al. performed a reliability scheduling algorithm for the static segment of FlexRay on vehicle networks [6]. The paper proposes a fast reliability scheduling algorithm (FRSA) to improve the communication reliability of FlexRay [6]. The proposed method reduces the probability of transient faults in one clock cycle by using a retransmission mechanism to recover the transient errors and further improves the computational complexity by using the lookup table method to ensure system reliability [6]. This paper analyzed the related literature to establish the system reliability constraints needed to evaluate the necessary time and slot usage, and the proposed cost function was used to evaluate the performance and efficiency when the number of messages was increased [6]. The experimental results show that the proposed FRSA reduces the execution time by an average of 70.76% and the cost by an average of 13.33% more than the other existing methods [6]. This method can be useful to others, especially regarding research about periodic time-triggered communication systems [6].

The studies [7,8] focused on the development of emerging sensing circuits. Lin focused on the fabrication and characterization of a high-performance multi-annular backscattered electron detector for desktop SEM [7]. The paper presents a silicon p-n diode with a multi-annular configuration to detect backscattering electrons (BSE) in a homemade desktop scanning electron microscope (SEM) [7]. The multi-annular configuration enables the enhancement of the topography contrast of 82.11 nA/μm as compared to the commercial multi-fan-shaped BSE detector of 40.08 nA/μm [7]. Additionally, the scheme integrated it with lateral p-n junction processing and an aluminum grid structure to increase the sensitivity and efficiency of the multi-annular BSE detector, giving a higher sensitivity of the atomic number contrast and a better surface topography contrast of BSE images for low-energy detection [7]. The responsivity data also show that MA-AL and MA p-n detectors have a higher gain value than the MA detector does [7]. The standard deviation of measurements is no higher than 1% [7]. These results verify that MA p-n and MA-AL detectors are stable and can function well in SEM for low-energy applications [7]. It is demonstrated that the multi-annular (MA) detectors are well suited for imaging in SEM systems [7].

Wang et al. design a novel dual-band six-phase voltage-control oscillator [8]. The voltage-controlled oscillator (VCO) with a single-ended delay cell architecture has a lower power consumption, a smaller chip area, and a larger output swing than one with a differential delay cell architecture [8]. However, the conventional even-phase output ring-type VCO cannot be implemented using single-ended delay cells [8]. In other words, the VCO with single-ended delay cells meets most of the requirements of a sensor circuit system, except for the even-phase outputs function [8]. This work presents a dual-band six-phase ring type VCO, which is implemented using the proposed single-ended delay cell [8]. The proposed VCO exhibits both the advantages of single-ended delay cells and differential delay cells [8]. The proposed delay cell has a band-switching function, which improves the jitter performance of the VCO in which it is used [8]. The proposed VCO can be operated at 890–1080 MHz [8]. The peak-to-peak jitter and the root mean square jitter are 35.5 ps and 2.8 ps (at 1 GHz), respectively [8]. The maximal power consumption is approximately 6.4 mW at a supply voltage of 1.8 V in a United Microelectronics Corporation 0.18 μm RF CMOS process [8]. The area of the chip is 0.195 × 0.208 mm^2^ [8].

Forecasting the future, in addition to the above eight key sensing technologies, there are still many key technologies and applications. The next generation of electronic technology will also focus on autonomous vehicles [9,10], medical electronics [11], artificial intelligence sensors [12], and sustainable development goals (SDGs) [13] related to sensing components, circuits, and systems. The diversity of applications in this Special Issue demonstrates the importance of novel research on emerging sensing and electronics technologies. Advanced sensing technology will lead the next generation of electronics to a new era and a better future.
